# Bending rod is unnecessary in single-level posterior internal fixation and fusion in treatment of lumbar degenerative diseases

**DOI:** 10.1186/s12893-021-01386-x

**Published:** 2021-11-06

**Authors:** Xiao Han, Xin Chen, Kuan Li, Zheng Li, Shugang Li

**Affiliations:** Department of Orthopaedics, Peking Union Medical College Hospital, Chinese Academy of Medical Sciences and Peking Union Medical College, Dongcheng District Shuaifuyuan No. 1, Beijing, 100730 China

**Keywords:** Orthopedic surgical procedures, Single level, Fusion, Fixation

## Abstract

**Background:**

Bending rod is a routine in lumbar fusion and fixation surgery, but there is no study investigating whether bending rod in one level is necessary.

**Methods:**

Patients receiving 1 level lumbar fixation and fusion between May 2018 and September 2020 were included with a minimum 6-month follow-up. The routine of bending rod was omitted during fixation. Preoperative and postoperative radiological parameters were compared.

**Results:**

There were 67 patients included in the study. Segment lordosis angle increased obviously from 10° (1–39°) to 14° (2–30°) immediately after operation (p = 0.000). T5-T12 increased from 22.97 ± 12.31° to 25.52 ± 11.83° by the 3rd months after surgery (p = 0.011). SS decreased from 35.45 ± 10.47 to 32.19 ± 11.37 in 6-month follow-up (p = 0.038), and PI dropped from 56.97 ± 14.24 to 53.19 ± 12.84 (p = 0.016). ROM of SLA decreased from 4.13 ± 3.14° to 1.93 ± 1.87° at that time point (p = 0.028). Those changes were not seen at 12-month follow-up. No evidence of adjacent vertebral disc degeneration was observed at any time point.

**Conclusions:**

No sagittal imbalance, dynamic instability or adjacent vertebral degeneration was observed by the 12th month after single-segment posterior lumbar fusion with the use of unbent rods. Bending rod could be omitted in 1-level lumbar fusion to simplify the procedure and reduce operating time.

## Background

Posterior internal fixation and bone graft fusion remains the gold standard for treatment of lumbar degenerative disc diseases (DDD). Despite of a variety of surgical approaches and fusion techniques, satisfactory fusion rate could never been realized without rigid fixation [1]. The classical fixation construct consists of pedicle screw and titanium rod. Due to physiological lumbar curvature in the sagittal plane, the straight rods are always bent to curve to simulate the lumbar lordosis angle (LLA) [2].

LLA is a component of sagittal spino-pelvic parameters, the restoration of which plays a key role in maintaining balance and preventing adverse events such as long-lasting postoperative low back pain, failure and adjacent vertebral diseases (AVD) [3, 4]. Previous studies focused mainly on the significance of bending rod in multi-levels lumbar fusion [5]. It is a general trend to cater the natural curve of lumbar spine without spending too much time preparing the bend rod, and computer-assisted pre-bent system has been developed to achieve this goal [6].

However, what is yet unclear is whether this procedure is meaningful for one-level surgery. Single-segment operations accounted for over one third of lumbar surgeries [7]. Extensive research has shown that one-segment lumbar fusion often brings about better clinical outcomes when compared with long-level counterparts [8]. It has been previously observed that one-level patients tend to spend less time in turning back to life and sport [9]. But several researchers have demonstrated that short-segment fixation also has considerable risk of AVD [10], so local changes of this surgery deserves special attention. Most of surgeons regard rod bending as an inherent step in posterior lumbar surgery, and do it mechanically without considering how much could one level’s lordosis angel contribute to the whole and part balance [11].

Our medical team has applied unbend rod as the fixation instrument in 1-level lumbar operations for years and has achieved great clinical outcomes so far, but there has never been a research to evaluate postoperative changes in imaging. This research is intended to reveal the overall and local difference between preoperative and postoperative radiological images. The index to be showed includes sagittal spino-pelvic parameters, and static and dynamic changes in responsible and adjacent segments. This would be a frank and straightforward response to queries about rationality and security of this simplified surgical procedure, as well as a reliable evidence to fill the gap in literature and enhance our understanding of mono-level lumbar fixation and fusion.

## Materials and methods

### Source of patients

The data were collected from patients underwent single-segment posterior lumbar fixation and fusion surgery in Orthopedics Department of Peking Union Medical College Hospital from May 2018 to September 2020. All operations were finished by one chief physician with 30-year clinical experience.

### Inclusion and exclusion criteria

Inclusion criteria were: (1) undergoing primary lumbar fixation and fusion surgery due to single-level DDD with sufficient indications for surgical intervention, including lumbar spinal stenosis (LSS), lumbar disc herniation (LDH), lumbar spondylolisthesis and spondylolysis; (2) being available for at least once follow-up in 3, 6 and 12 months postoperatively; (3) being willing to sign written informed consents to approve data collection and publication. Exclusion criteria include: (1) multi-levels fixation or pure discectomy without fixation. (2) vertebral fracture or other acute injuries; (3) combined with severe spinal deformity that need correction, or any diseases that may cause trunk imbalance; (4) revision or history of lumbar surgery in other segments; (5) lost to follow-up in all time points.

### Surgical procedure and postoperative rehabilitation

After checking the patient’s information and marks of surgical area, preoperative prophylactic antibiotics (cefuroxime 1.5 g, intravenous) was used and intratracheal general anesthesia was applied. The surgical area was disinfected and draped for sterility and the surgery was performed in a prone position. Following careful stripping of the paraspinal muscles, spinous process, bilateral articular processes and roots of transverse processes were exposed. Afterwards, titanium polyaxial pedicle screws (Legacy, Medtronic, USA) were inserted bipedicularly from the superior to inferior vertebrae. Then two unbent titanium rods were cut to appropriate length and placed between nuts, which are subsequently tightened to lock the rods. Afterwards, specific process like decompression, vertebra body reduction, articular fusion and interbody fusion are carried out according to different diagnosis.

After surgery, the volume of drainage was recorded every day, and the drainage tube was removed once wound drainage decreased down within 100 mL per day. Then patients were guided to stand and walk with the protection of individual- customized lumbar brace. They were asked to lie in bed for most of the day within one month after operation, and were encouraged to walk afterwards. By the third month after surgery, the lumbar brace was taken off, and a standard procedure including lumbar floating over, crouching, bending down and jogging was taught to strengthen their lumbar-dorsal muscles.

### Data collected

**Fig. 1:**
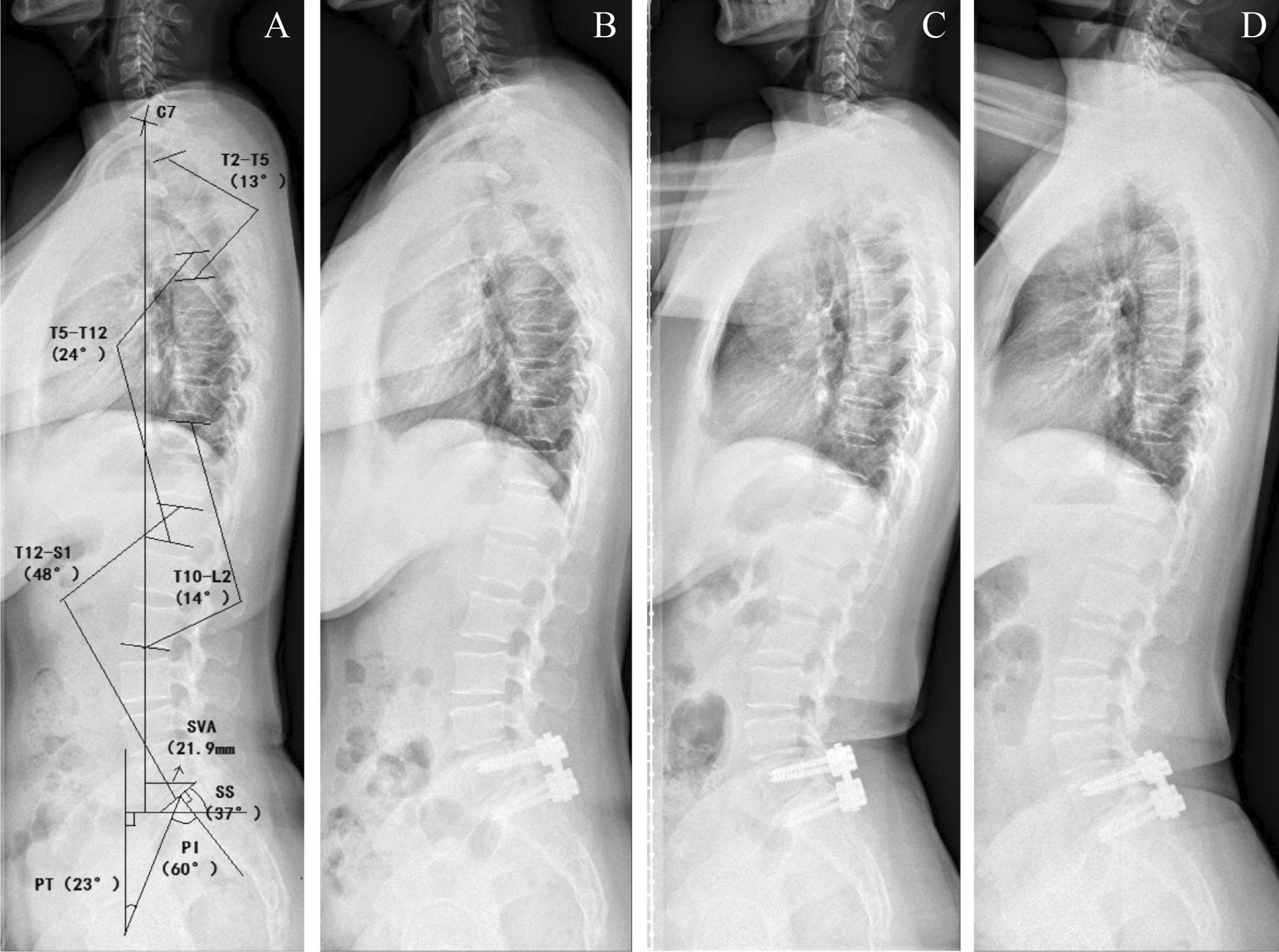
Lateral all-spine radiograph of baseline, 3-month, 6-month and 12-month follow-up. **A** Preoperative lateral all-spine radiograph. The method of measuring T2-T5, T5-T12, T10-L2, T12-S1, SS, PT, PI and SVA is shown in picture. **B** 3-month lateral all-spine radiograph. **C** 6-month lateral all-spine radiograph. **D** 12-month lateral all-spine radiograph

**Fig. 2:**
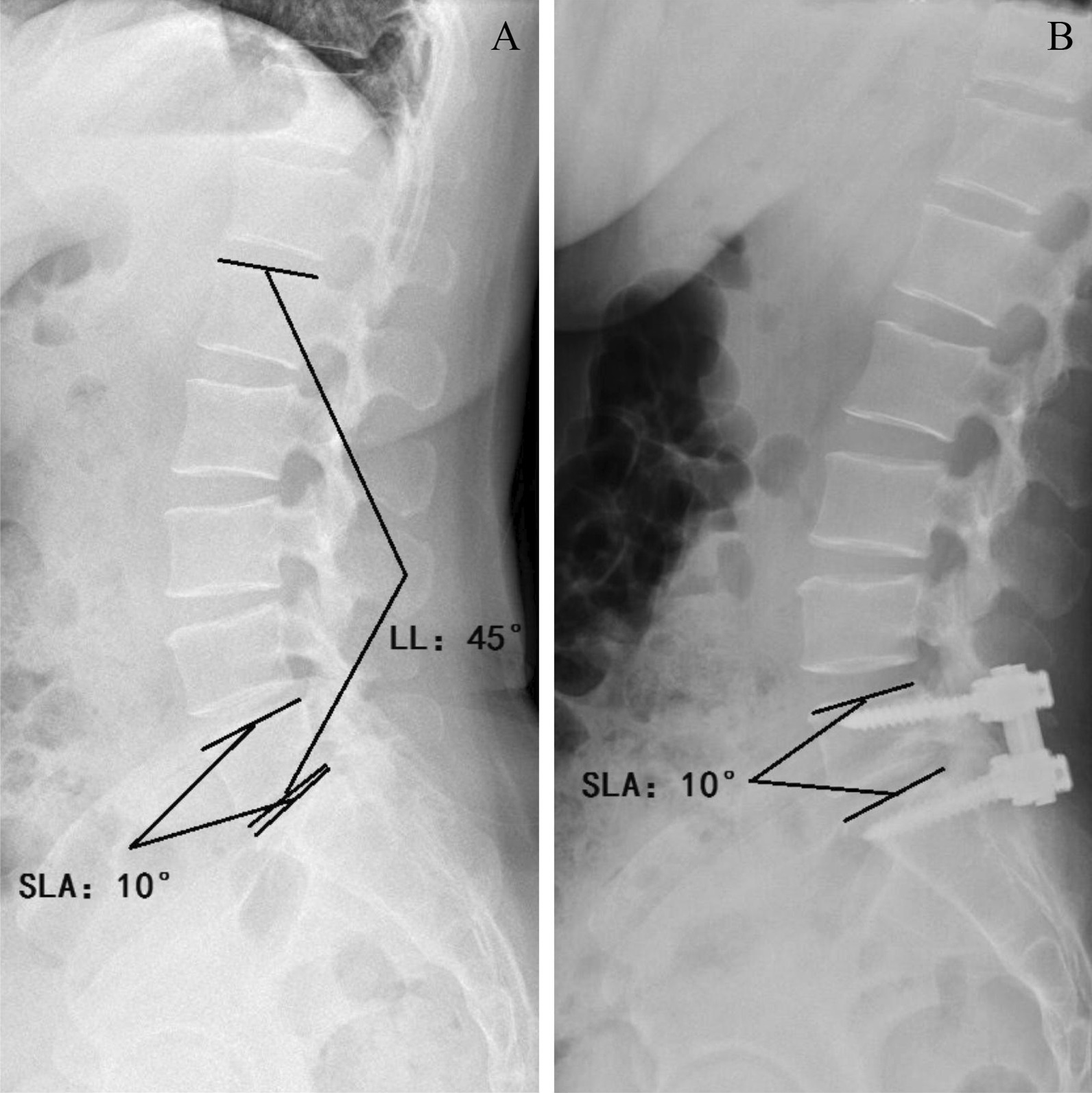
Preoperative and immediate postoperative lateral lumbar radiograph. **A** Preoperative lateral lumbar radiograph. The method of measuring SLA and LL is marked in picture. **B** Immediate postoperative lateral lumbar radiograph

**Fig. 3:**
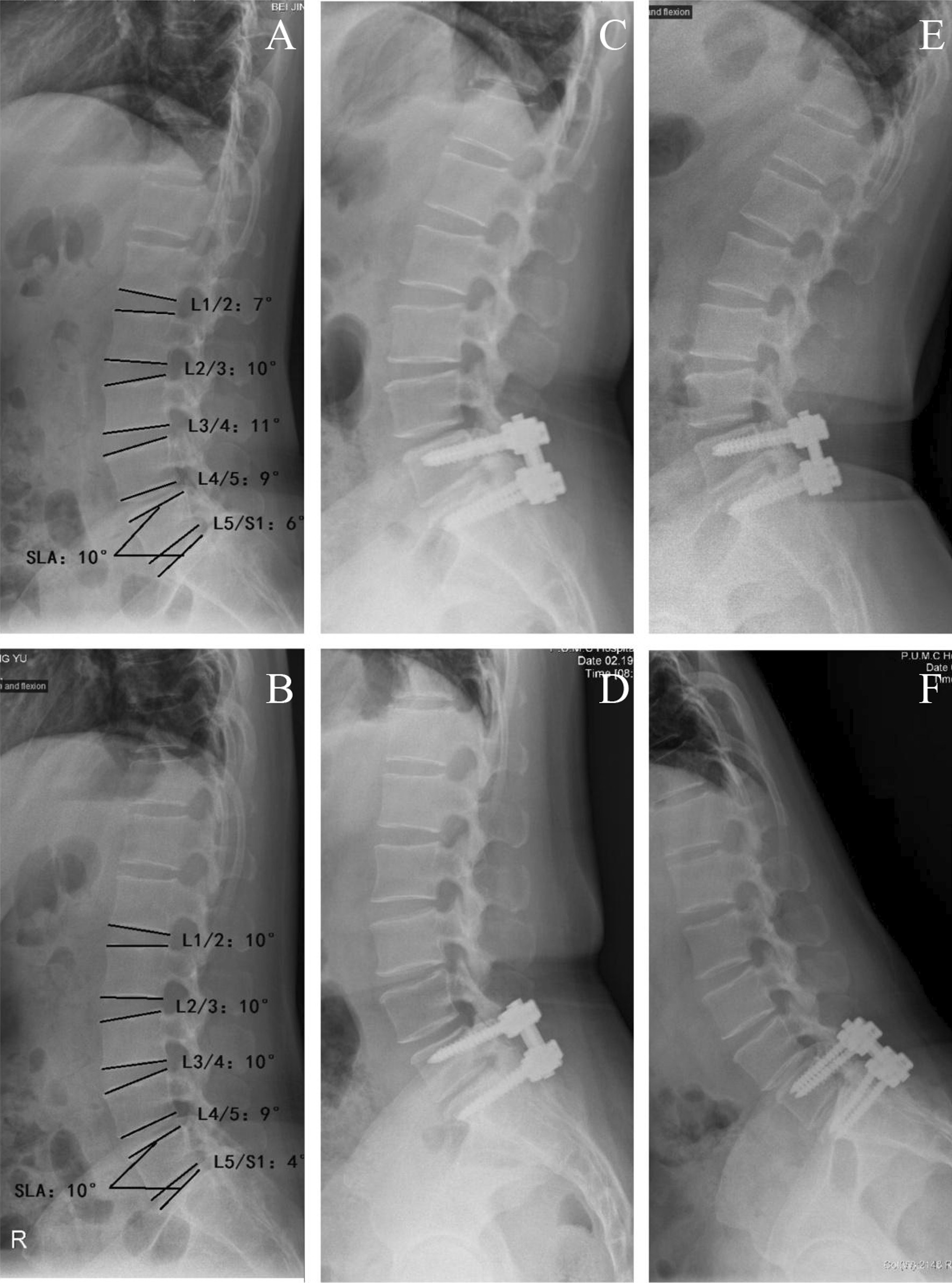
Preoperative, 6-month and 12-month lateral lumbar extension-flexion radiograph. **A** and **B** Preoperative lateral lumbar extension-flexion radiograph. Measurement of intervertebral angle and segmental lordosis angle are demonstrated in the picture. △intervertebral and ROM-SLA = extension-flexion. **C** and **D** 6-month lateral lumbar extension-flexion radiograph. **E** and **F** 12-month lateral lumbar extension-flexion radiograph

**Fig. 4:**
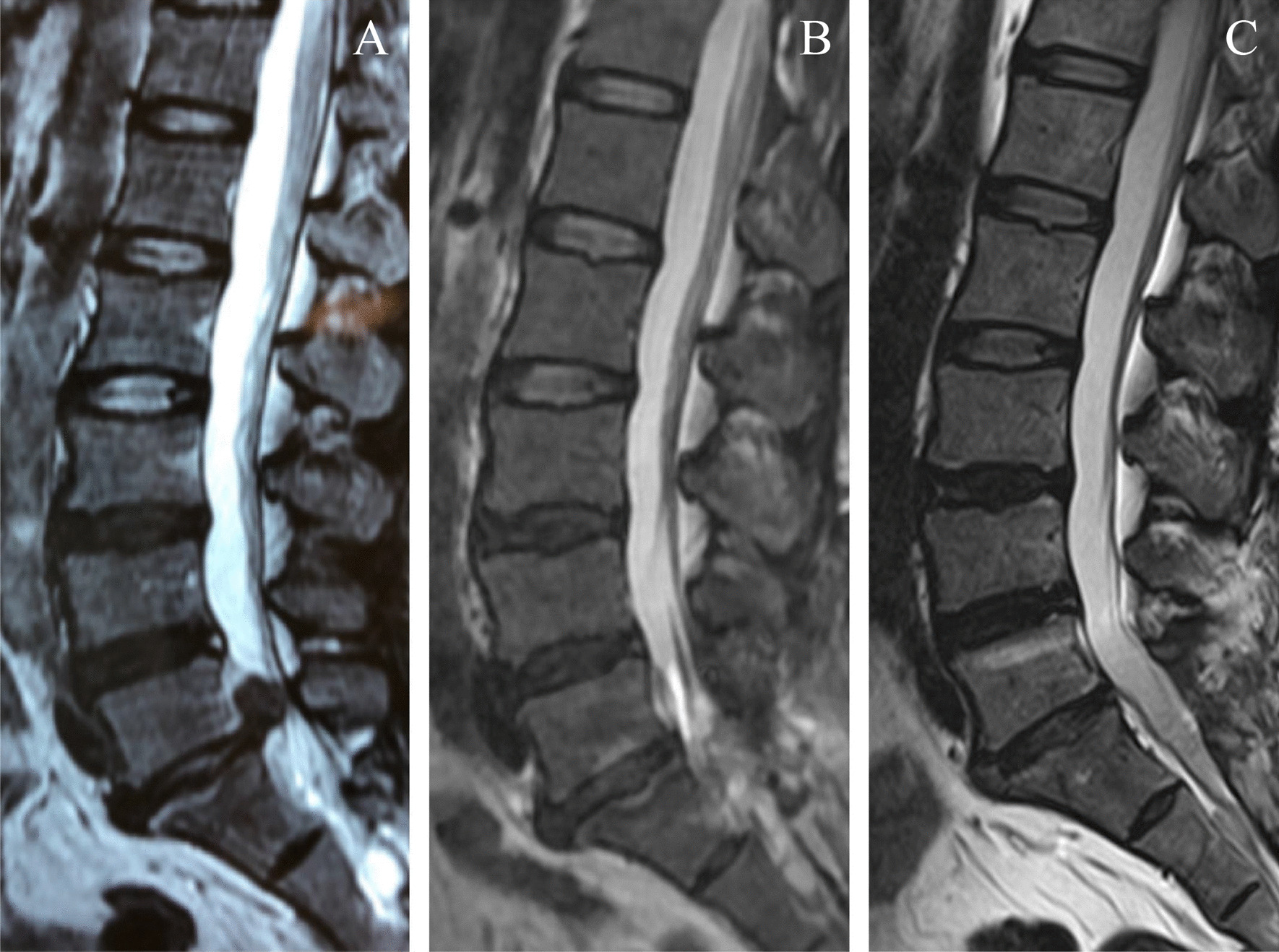
preoperative, 6-month and 12-month sagittal lumbar T2 weighted MRI. **A** Preoperative sagittal lumbar T2 weighted MRI. Pfirrmann level: 3(L1/2), 3(L2/3), 5(L3/4), 5(L4/5), 6(L5/S1). **B** 6-month sagittal lumbar T2 weighted MRI. Pfirrmann level: 2(L1/2), 2(L2/3), 5(L3/4), 5(L4/5), 7(L5/S1). **C** 12-month sagittal lumbar T2 weighted MRI. Pfirrmann level: 4(L1/2), 4(L2/3), 5(L3/4), 5(L4/5), 6(L5/S1)

Clinical data including patients’ general information and imaging findings were recorded. Preoperative imaging data include all-spine lateral radiograph, extension-flexion lateral radiograph and lumbar MRI. Immediate postoperative examination is lumbar lateral radiograph. In 3-month, 6-month and 12-month postoperative follow-up, patients accepted all-spine lateral X-Ray. Extension-flexion lateral radiograph and lumbar MRI was performed only in 6-month and 12-month follow-up. This arrangement is based on consideration of economic burdens on patients, time required for bone fusion, as well as suitable time to observe adjacent disc degeneration. Figure 1 shows the data we collected from lateral all-spine radiograph, as well as the details of measurement. Figure 2 is about what we were intended to analyze from preoperative and postoperative lateral lumbar radiograph. Special examinations including lateral lumbar extension-flexion radiograph and lumabr MRI were applied to evaluate the range of motion and adjacent vertebral conditions, which are demonstrated in Figs. 3 and  4 respectively.

All sagittal spino-pelvic parameters, including sagittal vertical axis (SVA), sacral slope (SS), pelvic tilt (PT), pelvic incidence (PI), upper thoracic kyphosis angle (T2-T5), lower thoracic kyphosis angle (T5-T12), thoracolumbar lordosis angle and lumbar lordosis angle (T12-S1) were recorded to describe the sagittal balance. The measurement standard of these parameters has been provided in previous articles [12].

Throughout this dissertation, the term “segment lordosis angle (SLA)” will be used to refer to the angle formed between upper endplate of superior responsible vertebrae body and the upper endplate of inferior responsible vertebrae body. This definition was made for two reasons. Firstly, the upper and lower endplate are not always parallel to each other because of possible wedging deformity of vertebrae body. Also, our team inserts pedicle screws strictly along a tunnel that parallel to the upper endplate, so SLA could represent the angle between upper and lower screws simultaneously. Preoperative and immediate, 3-month, 6-month and 12-month postoperative SLA was recorded to evaluate the curvature of surgical region. Intervetebral angle refer to angle between lower enaplate of superior vertebrae and upper endplate of inferior vertebrae. To differentiate the adjacent and responsible vertebrae, we use △ to describe the gap of intervertebral angle between extension and flexion position, and use range of motion (ROM) to represent the gap of SLA between extension position and flexion position. ROM and △ were recorded to show the changes in flexibility in 6-month and 12-month follow-up.

Degeneration of adjacent intervertebral disc is evaluated in T2 weighted sagittal lumbar MRI images. To assure the consistency of grading standard, all the images were the section in middle of lumbar disc. Pfirrmann index was used to grade the degree of degeneration from level 1 to 8 [13].

### Statistical methods

Data of each follow-up time point is compared with preoperative baseline to determine whether there is any difference. Above all, Shapiro–Wilk normality test was performed to judge the normality of all data. Normally distributed data were tested with the paired t-test and shown in the form of mean (standard deviation). Data that were not normally distributed was shown as median (range), and was tested by nonparametric Wilcoxon rank-sum test for pair comparisons. Statistical analysis was performed using SPSS software (version 20). The difference was considered statistically significant when P < 0.05.

### Statement

Ethics approval was obtained from ethics committee of Peking Union Medical College Hospital, and written informed consent for participation was obtained from the patient. All methods were performed in accordance with the relevant guidelines and regulations of Peking Union Medical College Hospital.

## Results

### General information

There were 103 single-segment surgeries in total. After excluding those fail to meet inclusion criteria, 67 cases were included in our investigation (28 males/39 females), with a mean age of 53.51 years old (53.51 ± 16.07), ranging between 12 and 81 years old. Most of cases were L4/5 lumbar fusion, accounting for 56.7% (38 cases), followed by L5/S1 (26 cases, 38.80%). The proportion of LSS, LDH, lumbar spondylolisthesis and spondylolysis was 55.2%, 31.3%, 47.8% and 25.4% respectively. It should be noticed that some of the patients had multi-diagnosis. Discectomy and inerbody fusion was applied in 21 (31.3%) and 28 (41.8%) cases respectively. In 3-month follow-up, we had 60 cases available for radiological examination. This figure dropped to 31 by 6 months postoperatively and then 27 in 12-month follow-up.

### Results immediately after operation

We compared preoperative and immediate postoperative SLA. Those data were not normally distributed and were tested by Wilcoxon rank-sum test. Median of SLA was 10° preoperatively, with a range from 1 to 39°. This figure increased to 14° (range: 2 to 30°) postoperatively. The difference was statistically significant (p = 0.000). This is the only parameter at this time point and is not shown in the Table.

### Results at 3-month follow-up

As shown in Table 1, T2-T5, T10-L2, SVA did not follow a normal distribution and were tested by Wilcoxon rank-sum test. Other parameters were tested by paired t-test. There was no significant difference in SVA, T2-T5, T10-L2, T12-S1, SS, PT and PI between preoperative and 3-month postoperative data (P > 0.05). T5-T12 increased in 3 months after surgery from 22.97 ± 12.31° to 25.52 ± 11.83° (p = 0.011).

**Table 1:** Sagittal physiological curvature and spinopelvic parameters at 3, 6 and 12-month follow-up

	3-month (N = 60)			6-month (N = 31)			12-month (N = 27)		
	Pre	Post	P	Pre	Post	P	Pre	Post	P
T2-T5 (°)	11 (0–38)	9.5 (0–31)	0.385	11.39 (7.43)	9.95 (6.38)	0.250	11 (0–38)	13 (0–35)	0.754
T5-T12 (°)	22.97 (12.31)	25.52 (11.83)	0.011	24.58 (11.67)	26.9 (9.79)	0.109	24.30 (12.11)	25.89 (11.26)	0.330
T10-L2 (°)	6 (0–49)	7.5 (1–49)	0.544	6 (0–19)	7 (0–22)	0.380	6 (0–49)	6 (0–53)	0.217
T12-S1 (°)	45.65 (15.80)	44.93 (11.66)	0.698	49.13 (14.81)	46.26 (13.61)	0.268	47.52 (13.10)	47.22 (14.29)	0.871
LL (°)	33.17 (12.04)	32.75 (11.11)	0.744	37.10 (11.03)	34.71 (13.16)	0.262	34.60 (12.58)	35.22 (14.30)	0.752
SS (°)	34.90 (10.59)	32.75 (9.12)	0.086	35.45 (10.47)	32.19 (11.37)	0.038	34.70 (8.81)	35.44 (9.11)	0.593
PT (°)	20.72 (9.40)	20.30 (7.34)	0.676	21.19 (7.20)	21.00 (5.76)	0.894	19.67 (9.79)	19.56 (7.14)	0.955
PI (°)	55.72 (13.46)	53.38 (12.04)	0.077	56.97 (14.24)	53.19 (12.84)	0.016	55.11 (11.95)	55.00 (10.77)	0.959
SVA (mm)	10.15 (−58.6–133)	7.4 (−40–69.3)	0.412	11.1 (−58.6–80)	12.38 (−24–73.7)	0.829	16.09 (30.67)	12.70 (25.74)	0.614

Pre, preoperative data

Post, postoperative data

T2-T5, Angle between upper endplate of T2 and lower endplate of T5

T5-T12, Angle between upper endplate of T5 and lower endplate of T12

T10-L2, Angle between upper endplate of T10 and lower endplate of L2

T12-S1, Angle between upper endplate of T12 and upper endplate of S1

LL, Angle between upper endplate of L1 and lower endplate of L5

SS, Sacral Slope

PT, Pelvic Tilt

PI, Pelvic Incidence

SVA, Sagittal Vertical Axis

Normally distributed data are shown as mean (standard deviation), and data that are not normally distributed are shown as median (range)

### Results at 6-month follow-up

From Table 1 we can find that SVA and T10-L2 in 6 months after operation were tested by Wilcoxon rank-sum test and other sagittal parameters were tested by paired t-test. It is shown in Table 1 that there was a significant drop in SS and PI by 6 months after operation. SS decreased from 35.45 ± 10.47 to 32.19 ± 11.37 (p = 0.038), and PI dropped from 56.97 ± 14.24 to 53.19 ± 12.84 (p = 0.016). Considering the formula of PI (SS + PT), this could be mainly explained by the decrease in SS. Other over-all sagittal parameters showed no difference.

At this time point, we also take the condition of responsible and adjacent vertebrae into consideration, and those patients were divided into L4/5 group and L5/S1 group (there was only one L3/4 patient available for follow-up, which was removed). Table 2 provides an overview of these data. In L4/5 group, no statistically significant difference in SLA was evident, but ROM of SLA decreased from 4.13 ± 3.14° to 1.93 ± 1.87° (p = 0.028). Adjacent IVD signal showed no obvious degenerative changes. No significant changes of those parameters above were detected in L5/S1 group.

**Table 2:** ROM of responsible and adjacent vertebrae and Pfirrmann score of adjacent intervertebral disc degeneration

Group		6-month			12-month		
		Pre	Post	P	Pre	Post	P
			(N = 15)			(N = 16)	
L4/5	△L3/4 (°)	4.33 (3.75)	2.27 (3.65)	0.182	1.31 (5.13)	4.31 (4.66)	0.125
	△L5/S1 (°)	3 (0–15)	1 (−3–5)	0.937	3.75 (6.89)	6.31 (8.62)	0.350
	SLA-L4/5 (°)	9.13 (4.41)	10.67 (4.22)	0.199	11.63 (6.34)	13.5 (4.20)	0.133
	ROM-SLA (°)	4.13 (3.14)	1.93 (1.87)	0.028	3.94 (3.15)	2.75 (3.45)	0.240

			(N = 14)			(N = 15)	
L4/5	Pfirrmann-L3/4(level)	5 (3–7)	4.5 (2–6)	0.257	5 (3–5)	5 (3–5)	0.655
	Pfirrmann-L5/S1(level)	5 (4–7)	5 (4–7)	0.317	5 (3–8)	5 (4–8)	0.366

			(N = 15)			(N = 11)	
L5/S1	△L4/5 (°)	5.07 (5.05)	5.13 (4.24)	0.194	2.64 (3.11)	5.00 (5.14)	0.179
	SLA(L5/S1) (°)	18.27 (9.25)	19.67 (7.19)	0.524	17.91 (10.76)	17.00 (9.83)	0.706
	ROM-SLA (°)	3.87 (6.52)	2.73 (2.46)	0.972	3 (−12–8)	1 (−7–5)	0.212

			(N = 14)			(N = 11)	
L5/S1	Pfirrmann-L4/5(level)	4.5 (2–8)	4 (3–8)	0.957	5 (1–8)	3.5 (2–8)	0.098

∆ Gap of intervetebral angle between extension position and flexion position (intervetebral angle = angle between lower endplate of superior vertebrae and upper endplate of inferior vertebrae)

SLA, segmental lordosis angle = angle between upper endplate of superior vertebrae and upper endplate of inferior vertebrae

ROM, range of motion

ROM-SLA, gap of SLA between extension position and flexion position

### Results at 12-month follow-up

At this time point, there was no significant in over-all sagittal parameters in comparison with preoperative data (Table 1). Also, there was no significant difference in responsible and adjacent vertebrae between baseline and follow-up data, neither in L4/5 nor L5/S1 group (Table 2).

## Discussion

This study found several radiological changes after single-level unbent rod fixation and fusion. Immediate postoperative SLA increased obviously. T5-T12 at 3-month follow-up was larger than figures at baseline. SS, PI and ROM of SLA decreased by the 6^th^ month after operation. Other parameters referring to physiological curvature, spinopelvic balance, motion of surgical and adjacent segment and degeneration of adjacent IVD did not see a significant variation.

The amount of segments involved in lumbar fixation and fusion surgery has a great influence on postoperative radiological and clinical outcomes. Single-level lumbar fusion is always reported with little change in over-all balance, local degeneration and clinical outcomes [14].

Immediate postoperative imaging showed an increase in SLA, which is a portion of LLA. A normal LLA is essential to maintain sagittal balance and reduce compressive and shoving force on vertebral body [15]. We have notice that many patients with DDD show a collapse of intervertebral space, and this could likely be more or less improved after internal fixation, especially for interbody fusion cases, as the geometry of cage could provide a lordosis angle. But in 3, 6 and 12 months after surgery, no significant difference in SLA was found. Not only SLA, but also the LLA did not witness a significant change, which is different from prior studies. Liu et al. reported that the degree of LLA reconstruction is primarily decided by the length of fusion, and surgeries ≥ 2 levels showed greater improvement in LLA [16]. In Liu’s opinion, long segment cases always have greater loss in LLA preoperatively, and reconstruction of this angle is therefore quite meaningful for them, especially for those combined with kyphosis. In this study, LLA of short-segment cases showed no significant changes by one year after operation, which corresponds to our results. But there was Mourad et al. found that 1-level lumbar fusion would not cause sagittal imbalance after a mean follow-up period of 35.1 months [17]. Lee’s study also showed that normal segmental and lumbar lordosis would not be changed by one-level posterior lumbar fusion [18]. As Stavros et al. have proposed, the effect of fixation and fusion instrument on sagittal parameter is quite weak after a long time follow-up, and this might be the consequence of self-adjustment and adaption. Long-term changes in SLA remain to be further discovered.

By the 3rd months after operation, the thoracic kyphosis angle of T5-T12 increased from 22.97 ± 12.31° to 25.52 ± 11.83° (p = 0.011). This result could not be explained by direct influence of surgery, and such change has only been reported in short-segment kyphosis correction [19]. We analyzed this phenomenon and propose it to be related to the lumbar brace. This is solid and inelastic protective clothing, which limits users’ lumbar flexion and extension motion. Due to low back pain in early time and fear of damage on incision, many patients tend to stand in an unconscious forward-leaning posture. The motion of thoracic-lumbar vertebrae is restricted by the brace, while flexion of upper thoracic vertebral is untrammeled, showing in the increase of angle between T5-T12.

In our study, patients’ SS and PI value has dropped obviously by 6th month postoperatively (from 35.45 ± 10.37° to 32.19 ± 11.37°, p = 0.038). Previous studies have investigated the influence of SS and PI changes on clinical outcome of lumbar fusion. According to Liow et al.’s latest research, patients diagnosed with lumbar degenerative disease would have lower incidence of low back pain if postoperative SS is over 30° [20]. In our study, both preoperative and postoperative SS are over 30°, so this change is not an indication of worse clinical outcomes. PI is the sum of SS and PT, and PI itself has been proved to be unrelated to negative results, such as AVD, fracture, screw pulling out and failure [21–23].

Another significant change is △L4-5 at 6-month follow-up, decreasing from 4.13 ± 3.14° to 1.93 ± 1.87° (p = 0.028). This change is not found at any follow-up period of L5/S1 group, or at longer follow-up of L4/5 group. It may results from psychological factors. The lateral flexion–extension radiograph is only carried on at 6-month and 12-month follow-up, and patients started to turn back to sport from 3rd month after operation. A possible explanation is that patients are afraid of acute injuries or potential damages on fixation instrument at that time, leading to nonstandard flexion and extension.

Other sagittal radiological parameters showed no significant changes. The possible reason is the balanced condition before operation leaved little space for improvement, as restoration of sagittal parameters is more likely to be found in patients with preoperative imbalance [24]. Previous study has found that after 5-years’ follow-up, even single-segment lumbar fusion showed obvious degeneration of adjacent IVD [25], but this is not observed in our research. Considering relatively shorter follow-up period of our research, further studies would be carried out to estimate this.

Our application of unbent rod is based on the design of fixation instrument. We chose Legacy polyaxial pedicle screw produced by Medtronic, which provides a motion range of ± 15° [26]. The median of SLA is 10 preoperatively and 14 postoperatively, which is within the motion range of the screw. It has been proved long ago that the polyaxial pedicle screw would not bring an adverse effect on the rigidity of fixation construct [27]. Therefore, the motion of screw is sufficient to provide the appropriate segment angle.

The omission of bending the rod is not just a simplification of surgical procedure, but also an advance in understanding of single-level lumbar fusion and minimally invasive surgery. It has been widely agreed that the reduction of operation time could minimize the rate of infection and other accidence related to surgery and anesthesia [28]. Rod bending is completed during operation, which requires surgeons to spend extra time to prepare. To ensure the consistency of bilateral rods, the process of bending is always repeated and adjusted for several times. The time spent on this action has never been calculated yet, but it has inevitably contributed to the delay of surgery. Moreover, due to the structure of rod-bending clamp, rod must be placed accurately in the groove, and this could be quite challenging for short rod because its length is even shorter than the groove. Even the most experienced spine surgeon has definitely witnessed the detachment and popup of rod during bending because of unbalanced press. This is very dangerous for both patient and surgeon because the kinetic energy of the rod is surprising and the direction of injection is irregular. Either a strike on anybody around the operation table or a springback after an infectious touch will be an unacceptable accidence. The purpose of bending rod is to reconstruct SLA and LLA, and this demands the symmetry of bilateral rods. However, rod bending is based on surgeon’s visual inspection and experience, so the quality control of bent rods is not always so reliable. In addition, LLA reconstruction should be based on the primary angle. For those who have suffered from DDD for years, lumbar kyphotic deformity may have existed for long and be adapted by bone, nerve roots as well as surrounding soft tissue [29]. An extensive distraction may achieve an ideal radiological curve, but bring about an unexplainable low back pain. Regulating the degree of LLA reconstruction is also difficult for handcraft bending [30].

A number of limitations need to be noted regarding the present study. First of all, the number of patients is small, which is mainly because of our strict inclusion and exclusion criteria. Also, this is a prospective innovation and a challenge to the traditional surgical habits. Although those positive results were negative by the 12th month after operation, this might be caused by the limitation of sample size. The current clinical results are encouraging, and we would carry on further follow-up and enlarge the scale of investigation to increase its reliability. Secondly, the condition of IVD in responsible segment is not fully investigated. This is due to the interference of discectomy and interbody fusion on the signal of IVD signal. The degeneration of responsible IVD is not so critical since the screw-rod system has undertaken most of pressure. In addition, lumbar spondylolisthesis and lumbar spondylolysis could affect sagittal parameters [31]. After enlargement of cases, we would divide patients into difference groups according to diagnosis, and the alignment of vertebral body would be taken into consideration. Furthermore, this is a self-control study instead of a random control trial. To guarantee the consistency of surgical procedure and follow-up, we choose cases only in our own medical team. All cases included were not combined with severe deformity that requires correction, so preoperative sagittal parameters could be regarded as balanced. This self-control study aims to determine whether the application of unbent rod fixation has any adverse influence on over-all and local sagittal balance and changes in adjacent vertebra. No evidence of imbalance or lost of motion or obvious degeneration have been shown in the longest follow-up, proving the reasonability and practicality of this surgical optimization. The lack of function evaluation and self-reported degree of satisfaction is also a limitation, but according to feedback in outpatient, most of patients showed excellent clinical outcomes. Such comparison in self-control study could only prove improvement after surgery, which is not so necessary.

## Conclusion

It is unnecessary to bend rods when placing fixation instrument during single-segment lumbar surgery. Despite of fluctuation of T5-T12 at 3-month follow-up and SS, PI and SLA ROM at 6-month follow-up, by the 12th month after operation, there are no significant adverse changes in the whole trunk curvature, sagittal spinopelvic balance, motion of responsible and adjacent vertebral body, degeneration of adjacent IVD. This modification of surgical process is worth promoting to reduce operation time and avoid accidence during operation.

## Data Availability

The medical records and original image used during this study are available from the corresponding author on reasonable request.

## Abbreviations

DDD: Degenerative Disc Diseases
LLA: Lumbar Lordosis Angle
AVD: Adjacent Vertebral Diseases
LSS: Lumbar Spinal Stenosis
LDH: Lumbar Disc Herniation
SVA: Sagittal Vertical Axis
SS: Sacral Slope
PT: Pelvic Tilt
PI: Pelvic Incidence
SLA: Segment Lordosis Angle
ROM: Range of Motion
